# Dental erosive wear and salivary flow rate in physically active young adults

**DOI:** 10.1186/1472-6831-12-8

**Published:** 2012-03-23

**Authors:** Aida Mulic, Anne Bjørg Tveit, Dag Songe, Hanne Sivertsen, Anne B Skaare

**Affiliations:** 1 Institute of Clinical Dentistry, University of Oslo, Oslo, Norway

**Keywords:** Dental erosion, diet, exercise, prevalence, saliva

## Abstract

**Background:**

Little attention has been directed towards identifying the relationship between physical exercise, dental erosive wear and salivary secretion. The study aimed i) to describe the prevalence and severity of dental erosive wear among a group of physically active young adults, ii) to describe the patterns of dietary consumption and lifestyle among these individuals and iii) to study possible effect of exercise on salivary flow rate.

**Methods:**

Young members (age range 18-32 years) of a fitness-centre were invited to participate in the study. Inclusion criteria were healthy young adults training hard at least twice a week. A non-exercising comparison group was selected from an ongoing study among 18-year-olds. Two hundred and twenty participants accepted an intraoral examination and completed a questionnaire. Seventy of the exercising participants provided saliva samples. The examination was performed at the fitness-centre or at a dental clinic (comparison group), using tested erosive wear system (VEDE). Saliva sampling (unstimulated and stimulated) was performed before and after exercise. Occlusal surfaces of the first molars in both jaws and the labial and palatal surfaces of the upper incisors and canines were selected as index teeth.

**Results:**

Dental erosive wear was registered in 64% of the exercising participants, more often in the older age group, and in 20% of the comparison group. Enamel lesions were most observed in the upper central incisors (33%); dentine lesions in lower first molar (27%). One fourth of the participants had erosive wear into dentine, significantly more in males than in females (p = 0.047). More participants with erosive wear had decreased salivary flow during exercise compared with the non-erosion group (p < 0.01). The stimulated salivary flow rate was in the lower rage (≤ 1 ml/min) among more than one third of the participants, and more erosive lesions were registered than in subjects with higher flow rates (p < 0.01).

**Conclusion:**

The study showed that a high proportion of physically active young adults have erosive lesions and indicate that hard exercise and decreased stimulated salivary flow rate may be associated with such wear.

## Background

Dental erosive wear is an irreversible condition of growing concern to dental practitioners and researchers. Recent publications have shown a high prevalence of erosive lesions in young individuals [1-3]. The severity of the condition depends on several factors, such as lifestyle and diet, type and time of exposure to an erosive agent, mineralization of dental tissue, and saliva composition [4]. Saliva is essential for the maintenance of oral health and decreased salivary flow causes a clinically significant oral imbalance [5]. Furthermore, diminished saliva production reduces the capacity to clear and neutralize dietary acids in the mouth contributing to erosive lesions in some individuals [6-8]. Järvinen et al. [6] found that patients with a salivary flow rate of ≤ 1 ml/min were at a five-time greater risk of developing dental erosions than those with higher flow rates.

An increased interest in "healthy" lifestyle involving regular exercise and healthy diet, can lead to dental problems such as erosive wear [7]. It is well-known that salivary flow rate and saliva's composition may be influenced by exercise [9,10], caused by rapid breathing and sweat-induced dehydration. As far as we know, there are no studies on a possible relationship between exercise, dental erosive wear and salivary secretions.

The aims of the present study were three-fold: i) to describe the prevalence and severity of dental erosive wear among a group of physically active young adults, ii) to describe the patterns of dietary consumption and lifestyle among these individuals and iii) to study possible effect of exercise on salivary flow rate.

## Methods

### Study population

The study involved 220 adults, 77 men and 143 women with an age range from 18 to 32 years (mean 21 years, SD 4). The sample of the adults was divided into two groups:

1) Exercise group: 104 participants (36 men, 68 women; age range 18-32; mean 25 years, SD 4) who worked out at a fitness centre twice or more per week. These participants were divided into two age groups: 18-25 years (n = 63; 17 men, 46 women; mean 22 years) and 26-32 years (n = 41; 19 men, 22 women; mean 29 years). All were non-smokers and free of any medications.

2) Comparison group: 116 individuals (41 men and 75 women, age 18 years), who attended the Public Dental Health Service (PDHS) for regular dental treatment and who were already participating in a study among Norwegian 18-year-olds. The inclusion criterion for these adolescents was no regular exercise during the last five years outside school.

Sample size calculation was performed prior to initiating the study, and showed that 120 participants were needed in each group to detect a difference between the two groups at a two-sided alpha level of 5% (type I error) and 80% power (type II error of 20%), when expecting 40% prevalence of erosive wear in the exercise group and 30% among the comparison participants.

### Exercise session

Each exercise session lasted between 60 and 90 minutes, and the equipment included stationary bike ergometers and treadmills.

### Clinical examination

In the exercise group, the examination was carried out at the fitness centre in a garden chair, using light, mouth mirror, dental probes and cotton rolls to dry the teeth. The comparison participants (controls) were examined as part of their regular dental visit at a PDHS clinic. The teeth were dried and, if necessary, cotton rolls were used to remove food debris. Sixteen surfaces per participant were examined: the occlusal surfaces of the first molars in both jaws and the labial and palatal surfaces of the upper incisors and canines. Dental erosive wear was classified by the Visual Erosion Dental Examination (VEDE) system [11], according to the following criteria: score 0: no erosion; score 1: initial loss of enamel, no dentine exposed; score 2: pronounced loss of enamel, no dentine exposed; score 3: exposure of dentine, < 1/3 of the surface involved; score 4: 1/3 - 2/3 of the dentine exposed; score 5: > 2/3 of dentine exposed. In cases of doubt the lower score was recorded. Only lesions that were considered as obvious dental erosive wear defects were registered, including cuppings/grooves of the molar cusps.

When index surfaces were filled, bonded with a retainer, considered to have attritions and wedge-shaped defects or the tooth was extracted, the surfaces and teeth were recorded as missing and excluded.

### Saliva collection

With the allocated resources and of convenience the first 70 participants arriving to the fitness centre were asked to provide the saliva samples in a quiet, isolated room. The participants were fully informed of the process of the saliva collection.

Prior to the exercise, the participants were told to relax in an upright sitting position for few minutes before collecting the unstimulated whole saliva. Immediately afterwards, they performed a standardized, 10 minutes collections of saliva by letting the saliva drip into a graduated plastic tube. After collecting the unstimulated saliva, the subjects were given an unflavoured paraffin gum to chew at a rate of their own chewing frequency for 5 minutes to collect the stimulated whole saliva. Swallowing was not permitted. After the collection, the amount of saliva (ignoring the foam) was measured to an accuracy of 0.1 ml and flow rate (ml/min) was determined for each saliva sample. The same process was repeated immediately after the exercise. The participants were instructed to consume liquid during exercise session as they normally would do.

### Questionnaire

In connection with the clinical intraoral examination, each participant was asked to complete a questionnaire. The questionnaire covered details of medical and dietary history and oral hygiene habits. The medical history included information about possible gastro-oesophageal reflux and type and frequency of any medication used regularly. The dietary questionnaire covered details of the frequency and quantity consumed of common drinks and foods associated with dental erosive wear such as orange/apple/grapefruit juice, carbonated beverages, sports drinks and some types of fruit like oranges, grapefruits and apples. Dental hygiene habits, the frequency and duration of tooth brushing, fluoride consumption and the time of last dental visit were also recorded.

### Reproducibility of scorings

To register the number and severity of dental erosive lesions, the exercise group was examined by the first author (AM), who had previously undergone training, calibration and examination using the VEDE system on both clinical intra-oral photographs and on a group of individuals [11,12]. The mean inter-examiner value was 0.77 (κ_w_) (on photographs) and 0.73 (κ_w_) (on patients) indicating substantial agreement [11,13]. Re-examination of the participants was not performed in the present study due to practical reasons. However, inthe earlier study [11], thirty 18 year-old adolescents (600 surfaces) were re-examined by the first author (AM) 10 to 21 days after their initial examination, indicating a very good level of agreement (κ_w _= 0.95) [13].

### Statistical analyses

The statistical analyses were performed using the Statistical Package for the Social Sciences (SPSS, Inc. Chicago, IL, USA version 16). The absolute frequencies and proportions were obtained for descriptive and bivariate analysis (Chi-squared test) to test for possible associations between the variables. The level of significance was set at 5%. The statistical analysis for the weighted kappa (κ_w_) was calculated using a spreadsheet programme (Microsoft Excel).

### Ethical considerations

The study was approved by the local Regional Committee for Medical Research Ethics and The Norwegian Social Science Data Services. Written, informed consent was obtained from all participants.

## Results

### Prevalence and distribution of dental erosive wear

**Figure 1:**
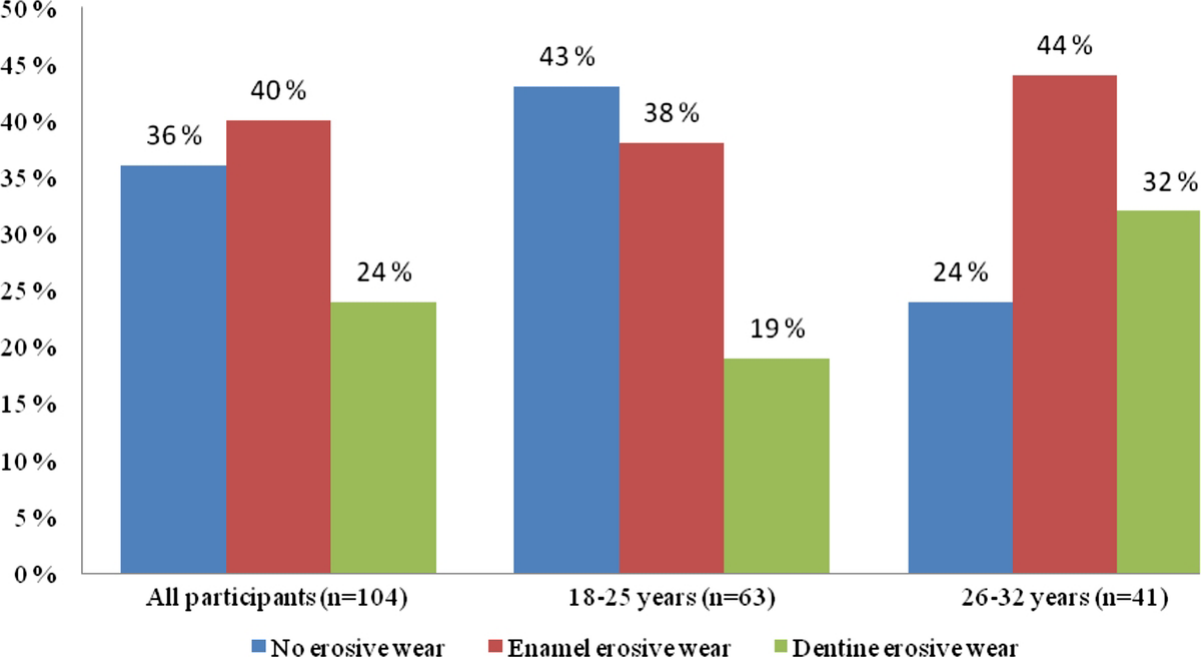
**Frequency and severity of dental erosive wear according to participants' age among physically active young adults (n = 104)**.

Dental erosive wear was registered in 64% of the exercising participants. In the age group 26-32 years, 76% had erosive lesions, while the prevalence was 57% among 18-25 year-olds (p < 0.01, Figure 1), higher than in the comparison group where 20% of the 18-year-olds had dental erosive wear (p < 0.01).

**Figure 2:**
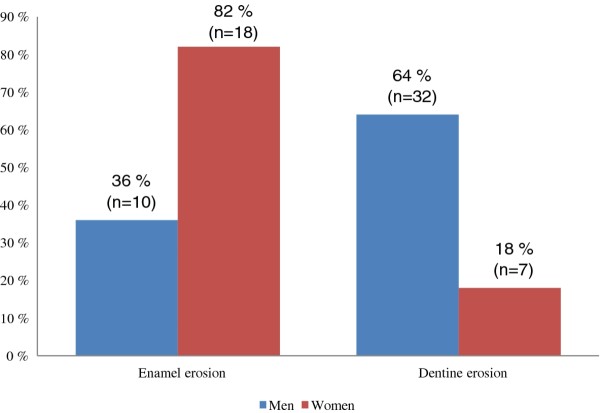
**Distribution of erosive wear in the exercise group according to sex**.

More men (78%) had erosive lesions than women (57%), but this difference was not statistically significant (p = 0.064). However, a significantly higher frequency of dentine lesions was found in men (p = 0.047; Figure 2).

No statistically significant differences were found between the numbers of lesions on contralateral tooth pairs. The highest frequency of erosive lesions was registered on the upper central incisors (33%), followed by first molars (27%). The majority of the lesions were confined to enamel. The highest occurrence of lesions with dentine involvement was found on the first molars (12%).

### Saliva collection

**Figure 3:**
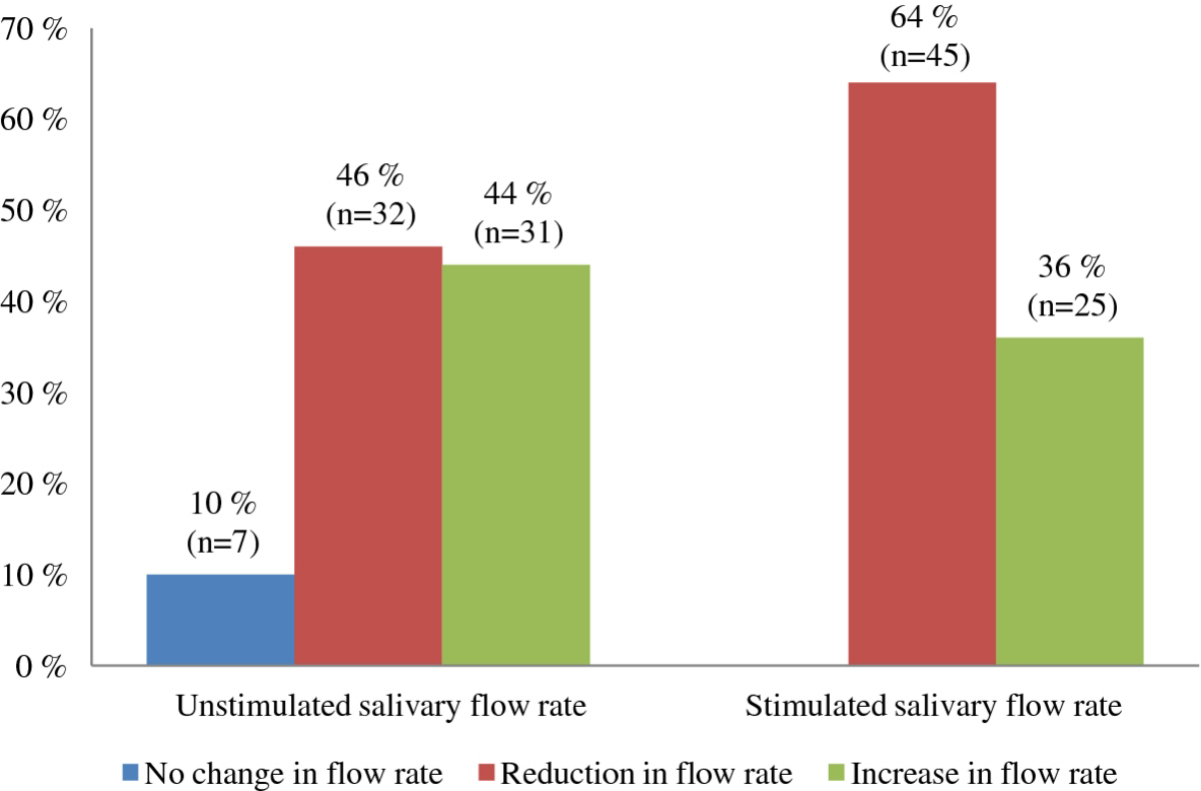
**Changes in unstimulated and stimulated salivary flow rate after exercise (n = 70)**.

In 64% (n = 45) of the individuals reduced stimulated salivary flow was registered after exercise whereas an increase was observed in 36%. The mean value before exercise was 1.43 ml/min (SD 0.09), while the mean value of 1.31 ml/min (SD 0.08) was measured after the training session. For the unstimulated saliva, nearly the same number of participants had reduced salivary flow (n = 32) as those who had an increased flow (n = 31) after exercise. In seven individuals (10%), the unstimulated flow rate remained unchanged (Figure 3). The mean value before exercise was 0.30 ml/min (SD 0.02), and 0.32 ml/min (SD 0.03) after exercise. A reduction in both stimulated and unstimulated salivary flow was registered in 36% (n = 25) of the participants, whereas 23% (n = 16) had an increase in both parameters. The remaining 41% (n = 29) had either an increase or a decrease in either unstimulated or stimulated salivary flow.

**Figure 4:**
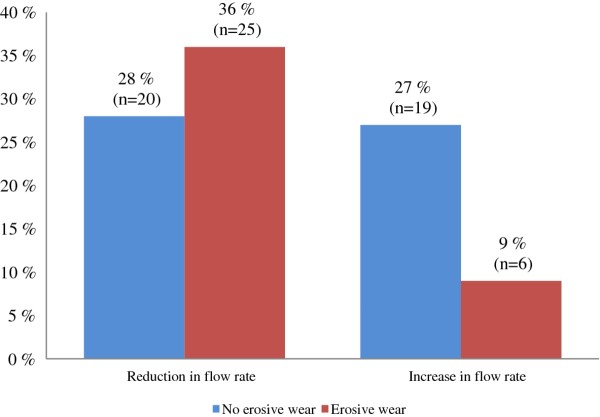
**Changes in stimulated salivary flow after exercise and prevalence of dental erosive wear**.

Of those with reduced stimulated salivary flow rate after exercise (n = 45), 36% had erosive wear, while of participants with increased salivary flow (n = 25), only 9% had erosive lesions (p < 0.01; Figure 4). Dentine lesions were registered more frequently among participants with reduced stimulated or unstimulated salivary flow compared with individuals with increased salivary flow (Table 1). Comparing the prevalence of erosive lesions among the "saliva providers" (n = 70) with the "non-saliva providers" (n = 44), no significant difference was observed.

**Table 1:** Distribution and severity grade of dental erosive wear among physically active young adults (n = 70)

	Unstimulated salivary flow			Stimulated salivary flow		
	No change	Reduction	Increase	No change	Reduction	Increase
	N (%)	N (%)	N (%)	N (%)	N (%)	
No erosive wear	3 (43)	15 (47)	8 (26)	20 (44)	19 (76)	

Enamel erosive wear	3 (43)	6 (19)	15 (48)	13 (29)	5 (20)	

Dentine erosive wear	1 (14)	11 (34)	8 (26)	12 (27)	1 (4)	

Table 2 shows distribution of stimulated and unstimulated salivary flow rates before and after exercise. Of the participants, 34% (before exercise) and 41% (after exercise) had stimulated salivary flow rate in the lower range (≤ 1 ml/min). The participants with stimulated and unstimulated salivary flow rate in the lower range had more erosive lesions than those with higher flow rates (p < 0.01).

**Table 2:** Distribution of unstimulated and stimulated salivary flow rates before and after exercise (n = 70)

Reference values	Unstimulated salivary flow		Reference values	Stimulated salivary flow	
	Before	After		Before	After
	N (%)	N (%)		N (%)	N (%)
≤ 0.1 ml/min	3 (4)	9 (13)	≤ 1 ml/min	24 (34)	29 (41)

> 0.1 ml/min	67 (96)	61 (87)	> 1 ml/min	46 (66)	41 (59)

### Questionnaire

#### Exercise session

Of the participants, 45% exercised 2-3 times per week, 37% 4-6 times per week, while 17% worked out daily. No statistically significant association could be observed between the presence of erosive wear and the amount of training (p = 0.90). During the exercise, all the participants reported consuming water, while three consumed sports drinks in addition.

#### Medical history

All participants in the exercise group were healthy adults, with no medical history. In the comparison group, 21 individuals (18%) used medications; but no dental erosive wear was seen in those participants.

Nearly one quarter (23%) of the individuals at the fitness centre reported the occurrence of gastro-oesophageal reflux and for 7% this was a weekly occurrence. No significant correlation between the occurrence of reflux and presence of erosive lesions could be observed. Only 4% reported reflux in the comparison group.

#### Dietary history

Consumption of acidic drinks and citrus fruits were dichotomized into high (once per day or more) and low (3-5 times per week or less) consumption. High consumption of acidic drinks was reported by 43%, while 23.5% had equivalent intake of acidic fruits (grapefruit, oranges, apples). Only 3% of the participants had a high consumption of sports drinks. No significant correlation between the intake of acidic drinks/fruits and the presence of dental erosive wear was found. The dietary questionnaire for the comparison group showed that 50% had a high consumption of acidic drinks; of these, 29% were registered with erosive lesions (p = 0.083). Furthermore, only 13% consumed fruits daily and all participants reported that they consumed sports drinks less than once per week.

#### Oral hygiene habits

Both groups of participants brushed their teeth twice a day for approximately 2 minutes. Among those who brushed more that 2 minutes, significantly more erosive wear was registered (p = 0.01). Only 19% of the individuals in both groups used daily fluoride rinses.

The participants in both groups reported regular dental visits with a time interval from 6 months up to 2 years. In the comparison group 66% and in the exercise group 63% had made their last dental visit not more than12 months prior to the examination. No statistically significant difference was observed between men and women regarding their last dental visit (p = 0.151). In the exercise group, 82% registered with dental erosive wear had not been informed by their dentist/dental hygienist about the presence of these lesions.

## Discussion

The present results revealed a higher prevalence of dental erosive wear among young physically active individuals compared with a group of young adults who did not exercise. A high consumption of acidic dietary components, such as beverages, citric fruits and sport drinks, as well as changes in salivary flow, have earlier been shown to increase the risk of erosive lesions [4,6,7,14-16]. In the present study, the questionnaire revealed a relatively high consumption of acidic beverages in both groups, particularly among the controls, but there was no significant association with erosive lesions. The consumption of citric fruits was relatively higher in the exercise group compared with the controls. Even though no association could be found with the erosive wear, the consumption may also be an explanation for the higher presence of lesions found among the individuals at the fitness centre. These findings suggest that isolating individual dietary components from other possible factors contributing to dental erosive wear may be too simplistic, and that the relationships between the factors leading to erosive lesions are complex. Furthermore, some studies have demonstrated that sports drinks used during exercise are not associated with erosive lesions in the athletes studied [14,17-19], whereas Järvinen [6] found a four-fold increase in risk of lesions when sports drinks were consumed. In the present study, consumption of sports drinks was not related to erosive wear. This could be explained by the small number of responders consuming sports drinks (only 3). As the participants were regularly undertaking exercise, but not necessarily competitively, they did not use nutrient replacements. In addition, the participants may have been aware of the fact that, for most individuals, the sports drinks offer no more benefits than water [19].

A higher prevalence of erosive wear in patients complaining of reflux symptoms have been reported [6,20,21]. In the study by Bartlett et al.[21], 64% of the patients with palatal erosion had pathological reflux symptoms. Although no significant association could be found in the present study, more than one fourth of the physically active participants reported occasions of reflux symptoms, a relatively higher frequency than reported in the comparison group. This indicates that physically active individuals may be at risk for development of erosive lesions which can be related to reflux symptoms. Previously, it has been noted that gastroesophageal reflux may be associated with some forms of tough exercise [22,23]. The study by Clark et al. [22] has shown that running and weightlifting induced reflux in healthy individuals, and that reflux persist through a 1-hour run.

While good oral hygiene is of proven value in the prevention of periodontal disease and dental caries, frequent tooth brushing may accelerate dental erosive wear [4]. It has been suggested that health-conscious individuals tend to have better than average oral hygiene [7]. The present study revealed that brushing teeth for more than two minutes at time was related to erosive lesions in both groups.

The questionnaire revealed that 82% of the physically active young adults with erosive wear who recently had been to their dentist/dental hygienist had not been informed about the presence of these lesions. This indicates a lack of awareness among dental practitioners regarding dental erosive wear and an increased risk for some physically active people who practice good oral hygiene.

The prevalence of dental erosion increases with age [24], because older individuals are more likely to have exposed their teeth to acidic diets for a longer time. The findings from the present study support this trend. The older age group (26-32 years) had a higher prevalence and more severe erosive lesions than participants in the age group 18-25 years.

However, the findings should be interpreted with caution since our study has some limitations. There were slightly more women than men among the cases, and the controls were on average four years younger. Furthermore, the conditions of the dental examination differ between the groups which could also have impacted our results. However, with no prevalence studies on dental erosive wear from Norway, and due to the difficulty of comparing studies from other countries because of different populations/age groups studied and examination standards, we decided to include a comparison group even though it was not perfectly matched. Furthermore, assessing the effects of acidic diet and other related factors based on questionnaires may not provide accurate data as the answers are limited by the respondents' ability to recall.

During physical activity, decreased stimulated salivary flow was observed among more than half (64%) of the participants. Earlier studies have demonstrated that saliva flow rate appears to be modified during exercise [9,10]. A decrease in salivary flow might be explained by an increase in sympathetic activity during intense exercise, since sympathetic innervations cause a marked vasoconstriction, resulting in reduced salivary volume [25]. This may also be a consequence of sweat-induced dehydration and restricted fluid intake during exercise. In a study by Horswill [16], a significantly lower stimulated salivary flow rate and volume was shown even when consuming water during the training session.

Prolonged exercise may reduce the unstimulated salivary flow [26]. Our results showed no consistency - the unstimulated salivary flow increased as often as it decreased among the participants. One could speculate that the duration of the training session was too short to give measurable changes in unstimulated saliva, since it has been suggested that modification of hydration status can at the earliest be detected three hours after exercise [27]. Another explanation of variability in the salivary flow rate may be individual variations [28], as well as consumption of fluids during the exercise [10,16]. Furthermore, by providing the saliva sample of only 70 out of 104 participants could have influenced the outcome. With the allocated resources and of convenience the first 70 participants arriving to the fitness centre were asked to provide the saliva samples. Comparing the prevalence of erosive lesions among the "saliva providers" with the "non-saliva providers", no significant difference was observed. Furthermore, there are no reasons to believe that the variations in flow rate between these participants should be different from the others. However, due to this uncertainty the results in the present study should be interpreted with caution. The participants consumed liquid during exercise session as they normally would with the intention to create a "real life situation" for the individuals. This could explain diversity in the unstimulated salivary flow rates and could have influenced the outcome of the present study, as liquid consumption during exercise may help maintain normal salivary function [16]. Another issue which could influence the salivary flow rates is diet and liquid intake before the exercise. It is known that previous stimulation of less than 1 hour prior saliva collection may influence the flow rate [29].

Several studies have demonstrated that reduced salivary flow may increase the risk to the dentition [4,6,7]. Järvinen et al. [6] found a low stimulated salivary flow in 16 erosion cases and 6 controls, while a reduction in unstimulated flow was seen in 7 erosion cases and 6 controls. These findings are in accordance with the present results. Although most participants studied demonstrated normal salivary flow rate, the stimulated salivary flow of more than one third was in the lower rage and significantly more erosive lesions were registered than in subjects with higher flow rates. Our findings support the statement of Järvinen et al. [6] that salivary flow rate is an important factor determining whether erosive lesions occur. One explanation could be the findings reported by Amaechi [30], higher salivary flow contributes to higher clearance and thus a lower erosive potential.

## Conclusion

The high prevalence of dental erosive wear reported reflects a need for preventive programmes and counselling for physically active young adults as it has been shown that exercise and decreased salivary flow rate may be two of many factors contributing to dental erosive wear. However, in order to implement adequate preventive strategies, further research is still necessary to clarify the etiology of erosive wear, focusing on the biological, chemical and behavioural factors involved.

## Competing interests

The authors report no conflicts of interest. The authors alone are responsible for the content and writing of the paper.

## Authors' contributions

AM carried out the data collection, assisted by DS and HS, data analysis and writing of the article. ABT initiated the idea and along with the ABS supervised the project and assisted in writing/editing of the article. All authors have read and approved the final manuscript.

## Pre-publication history

The pre-publication history for this paper can be accessed here:

http://www.biomedcentral.com/1472-6831/12/8/prepub
